# ^68^Ga-DOTATATE PET imaging in clinically non-functioning pituitary macroadenomas

**DOI:** 10.1186/s41824-020-0073-3

**Published:** 2020-02-27

**Authors:** Tessel M. Boertien, Jan Booij, Charles B. L. M. Majoie, Madeleine L. Drent, Alberto M. Pereira, Nienke R. Biermasz, Suat Simsek, Ronald Groote Veldman, Marcel P. M. Stokkel, Peter H. Bisschop, Eric Fliers

**Affiliations:** 1 Department of Endocrinology and Metabolism, Amsterdam UMC, University of Amsterdam, Meibergdreef 9, Amsterdam, the Netherlands; 2 Department of Radiology and Nuclear Medicine, Amsterdam UMC, University of Amsterdam, Meibergdreef 9, Amsterdam, the Netherlands; 3 Department of Internal Medicine, Section of Endocrinology, Amsterdam UMC, VU University, De Boelelaan 1117, Amsterdam, the Netherlands; 4 Department of Medicine, Division of Endocrinology, and Center for Endocrine Tumors Leiden (CETL), Leiden University Medical Center, Leiden, the Netherlands; 5 Department of Internal Medicine, Northwest Clinics, Alkmaar, the Netherlands; 6 Department of Internal Medicine, Medical Spectrum Twente, Enschede, the Netherlands; 7 Department of Nuclear Medicine, Netherlands Cancer Institute, Amsterdam, the Netherlands

**Keywords:** Non-functioning pituitary adenoma, Somatostatin receptors, ^68^Ga-DOTATATE, PET/CT, MRI co-registration

## Abstract

**Purpose:**

Clinically non-functioning pituitary macroadenomas (NFMA) have been reported to express somatostatin receptors (SSTR), but results are inconsistent across different studies. This may be related to limited sensitivity and specificity of techniques used to date, i.e. immunohistochemistry in surgical specimens and ^111^In-DTPA-octreotide scintigraphy in vivo. The aim of this study was to assess SSTR expression in NFMA in vivo using ^68^Ga-DOTATATE PET, which offers superior sensitivity and spatial resolution as compared with planar scintigraphy or SPECT.

**Methods:**

Thirty-seven patients diagnosed with NFMA underwent ^68^Ga-DOTATATE PET/CT of the head in the framework of a randomised controlled trial assessing the effect of the somatostatin analogue lanreotide on NFMA size. Individual co-registered T1-weighted pituitary MRIs were used to assess ^68^Ga-DOTATATE uptake (SUV_mean_) in the adenoma. An SUV_mean_ of > 2 was considered positive.

**Results:**

^68^Ga-DOTATATE uptake was positive in 34/37 patients (92%), with SUV_mean_ of positive adenomas ranging from 2.1 to 12.4 (mean ± SD 5.8 ± 2.6).

**Conclusions:**

This is the first report of ^68^Ga-DOTATATE PET performed in NFMA patients, demonstrating in vivo SSTR expression in the vast majority of cases. The high positivity rate when compared with results obtained with ^111^In-DTPA-octreotide scintigraphy probably reflects the superior sensitivity of PET imaging.

**Trial registration:**

Netherlands Trial Register, NL5136, registered on 18 August 2015; EudraCT, 2015-001234-22, registered on 10 March 2015, https://eudract.ema.europa.eu/

## Introduction

Pituitary adenomas are benign tumours of the pituitary gland and the third most common intracranial neoplasm. Around 30% are clinically non-functioning adenomas (NFA) that lack clinical and biochemical signs of hormonal activity (Even-Zohar and Greenman 2018). Due to the absence of signs related to hormonal hypersecretion, NFA usually present as macroadenomas (i.e. diameter ≥ 1 cm), causing compression of surrounding structures. Surgical resection is often incomplete, especially in the case of cavernous sinus invasion, and regrowth of residual adenoma tissue is common (Even-Zohar and Greenman 2018). Although adjuvant radiotherapy is effective in preventing tumour regrowth, its use is controversial because of long-term side effects such as hypopituitarism and neurocognitive dysfunction (Even-Zohar and Greenman 2018). This explains the ongoing interest in medical treatment, including somatostatin analogues (SSA). The rationale for this approach is the expression of somatostatin receptor (SSTR) subtypes in a varying proportion of NFA, as demonstrated by quantitative PCR and immunohistochemistry studies (Fusco et al. 2012; Ramírez et al. 2012; Gabalec et al. 2015). In vivo visualisation of SSTR expression via ^111^In-DTPA-octreotide planar scintigraphy or SPECT has shown increased uptake in about two thirds of patients (Fusco et al. 2012; Borson-Chazot et al. 1997). Encouraged by these findings, several open-label studies have investigated the effect of the SSA octreotide in NFA patients. Thus far, success has been modest, with tumour reduction in 12% of patients and no clear correlation with SSTR expression based on ^111^In-DTPA-octreotide scintigraphy results (for review, see Colao et al. 2008). However, the number of patients per study did not exceed 20, and the average follow-up period was only 6 months. One recent study with a mean follow-up of 3 years showed stable adenoma size in 81% of 26 patients, pre-selected through positive ^111^In-DTPA-octreotide uptake, compared with stability in 47% of 13 untreated patients that had negative uptake (Fusco et al. 2012).

In vivo evaluation of SSTR expression to predict clinical response to SSA is an attractive approach, especially since it does not require a surgical specimen and can thus be performed preoperatively. However, interpretation of pituitary adenoma uptake using planar scintigraphy/SPECT has several limitations, most importantly limited spatial resolution. PET/CT imaging with the SSA radiotracer ^68^Ga-DOTATATE provides better resolution and sensitivity and allows for accurate quantification of uptake within the adenoma (Bai et al. 2013; Aalbersberg et al. 2019). ^68^Ga-DOTATATE PET/CT could thus be superior to ^111^In-DTPA-octreotide scintigraphy for the assessment of SSTR expression in NFA. However, the rate of positive ^68^Ga-DOTATATE uptake in NFA has not been reported to date. This was the aim of the present study.

## Methods

### Patients

Adult patients diagnosed with a clinically non-functioning pituitary macroadenoma (NFMA) with suprasellar extension, either surgery-naive or as a postoperative remnant, were eligible for inclusion. Patients were referred by endocrinologists at hospitals in the Netherlands for inclusion at one of the participating centres (Amsterdam University Medical Centres (locations AMC and VUmc) and Leiden University Medical Centre) as part of a multicentre randomised controlled trial on the effect of lanreotide on NFMA size. NFMA diagnosis was based on neuroradiological evidence for a pituitary macroadenoma on MRI and absence of clinical and biochemical signs of hormonal overproduction. Exclusion criteria included visual field defects due to optic chiasm compression, previous radiotherapy involving the pituitary region and previous use of SSA. The study protocol was approved by the Medical Ethics Committee of the AMC and registered at the Netherlands Trial Register (NL5136) and EudraCT (2015-001234-22). All participants provided written informed consent.

### Imaging

Brain PET/CT imaging (Gemini ToF, Philips Medical Systems and Biograph mCT, Siemens Healthineers) was performed at the Netherlands Cancer Institute and the Amsterdam UMC, location AMC. ^68^Ga-DOTATATE radiosynthesis and quality control were performed as described earlier (Aalbersberg et al. 2019). Acquisitions were obtained approximately 45 min after intravenous bolus injection of 100 MBq ^68^Ga-DOTATATE with 2.5–3 min per bed position. A low-dose CT scan was acquired for attenuation correction and anatomical correlation (120 kVp, 60 mAs, pitch 0.813). Pituitary MRI was performed at the referring hospital as part of standard care on a 1.5- or 3-T scanner, following a pituitary-specific protocol that includes acquisitions before and after gadolinium administration and preferably a 3D T1-weighted sequence.

### Image analysis

PET/CT and MRI were co-registered using Hybrid Viewer (Hermes Medical Solutions, version 2.8.2). ^68^Ga-DOTATATE uptake in the adenoma was assessed on the fused PET/MR images by placement of a circular region of interest (ROI) within the adenoma on the coronal plane. The ROI covered the larger part of the adenoma diameter and was non-fixed to account for variability in adenoma size. A clear margin from normal pituitary tissue was maintained to avoid activity spillover effects from physiological pituitary uptake (Bai et al. 2013). ROI placement was performed by the same physician (TB). Within the defined ROI, the mean standard uptake value (SUV_mean_) was determined, which was confirmed by the same nuclear medicine physician in all patients (JB). Positive uptake was defined as an SUV_mean_ of > 2, based on a ^68^Ga-DOTATATE biodistribution study in which physiological pituitary uptake always had an SUV_mean_ of > 2 (Shastry et al. 2010).

### Statistical analysis

Continuous variables are reported as mean with standard deviation (SD). Categorical variables are expressed as proportions. Data were analysed using the SPSS software (IBM SPSS Statistics, version 25).

## Results

**Fig. 1:**
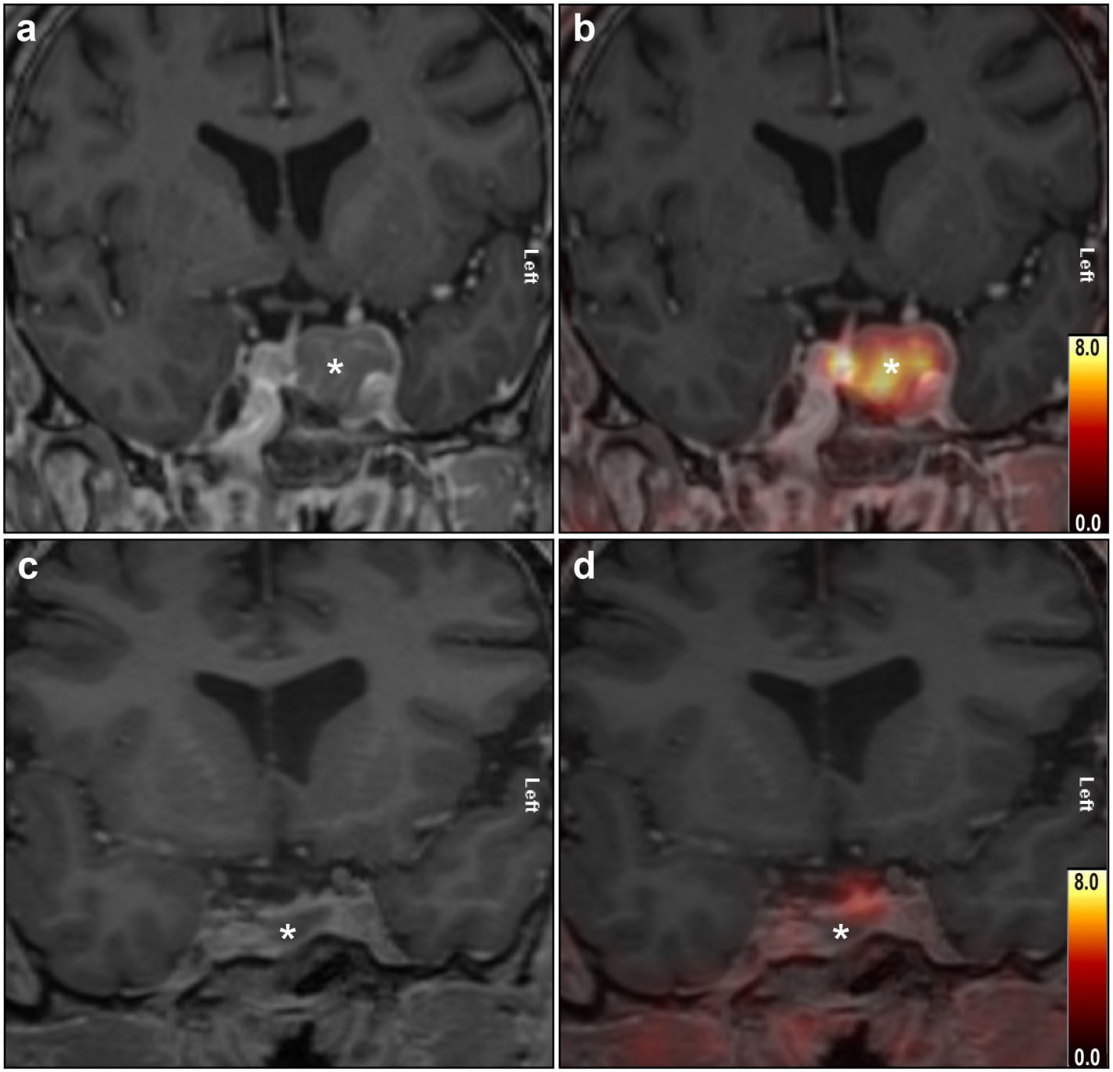
Coronal T1-weighted contrast-enhanced MRI and fused PET/MR images of a ^68^Ga-DOTATATE PET-positive (**a**, **b**) and PET-negative (**c**, **d**) NFMA. The colour bars represent SUV. **a** MRI shows a pituitary macroadenoma (asterisk) located laterally in the sella with an invasion of the left cavernous sinus. **b** After fusion with PET, intense uptake is seen in the NFMA (SUV_mean_ 5.7) as well as physiological uptake in the pituitary gland on the right side of the adenoma. **c** In this case, the largest part of the adenoma (asterisk) is located on the right side of the pituitary stalk. **d** The fused PET/MRI shows no uptake in the NFMA (SUV_mean_ 1.1) and moderate physiological uptake in the pituitary gland situated on top of the adenoma

Thirty-seven NFMA patients were included between November 2015 and July 2019. Adenoma size, measured as the maximum diameter in any direction, ranged from 12 to 44 mm. Clinical characteristics and ^68^Ga-DOTATATE PET uptake results are summarised in Table 1. ^68^Ga-DOTATATE uptake was positive in 34/37 patients (91.9%), with SUV_mean_ ranging from 2.1 to 12.4 (mean ± SD 5.8 ± 2.6). The 3 PET-negative patients had SUV_mean_ values of 0.7, 0.8 and 1.1. There was no significant correlation between adenoma size and SUV_mean_ (*r*_*s*_ = 0.186, *p* = 0.259, Spearman’s rank correlation). Because of the small number of PET-negative patients, no statistical tests were performed on group differences. However, clinical characteristics seemed to be comparable between patients with positive and negative uptake. ^68^Ga-DOTATATE PET-positive and PET-negative cases are presented in Fig. 1.

**Table 1:** Patient characteristics

Characteristics	All patients (*n* = 37)	^68^Gallium DOTATATE uptake	
		Positive (*n* = 34)	Negative (*n* = 3)
Age, years (mean ± SD)	59 ± 10	60.5 ± 9	43.3 ± 10
Sex, male (%)	25 (68%)	23 (68%)	2 (67%)
Maximum NFMA diameter (mean ± SD)	21 ± 5.6	21 ± 5.5	16 ± 4.6
Previous NFMA resection, no. (%)	19 (51%)	18 (53%)	1 (33%)
Pituitary hormone deficiency, no. (%), of which:	20 (54%)	18 (53%)	2 (67%)
ACTH deficiency	16	14	2
GH deficiency	6	4	2
TSH deficiency	12	11	1
LH/FSH deficiency	12	11	1
NFMA ROI SUV_mean_ (mean ± SD)	5.3 ± 2.8	5.8 ± 2.6	0.9 ± 0.2

*ACTH* adrenocorticotropic hormone, *FSH* follicle-stimulating hormone, *GH* growth hormone, *LH* luteinising hormone, *NFMA* non-functioning macroadenoma, *ROI* region of interest, *SUV* standard uptake value, *TSH* thyroid-stimulating hormone

## Discussion

In this study, we demonstrate positive ^68^Ga-DOTATATE uptake in 92% of NFMA (34/37). This positivity rate is higher than the two thirds of patients as reported earlier with ^111^In-DTPA-octreotide planar scintigraphy or SPECT (Fusco et al. 2012; Borson-Chazot et al. 1997). One likely explanation for this discrepancy is the superior sensitivity, higher spatial resolution and better partial volume recovery of PET (Bai et al. 2013). The limited resolution of planar scintigraphy/SPECT makes differentiation of ^111^In-DTPA-octreotide uptake in the adenoma from physiological uptake in the adjacent pituitary gland difficult. Most studies have therefore compared the uptake in the sellar region of patients to that of subjects without a pituitary disease, using either a visual grading system or a background-corrected uptake index to interpret results (Fusco et al. 2012). With this method, only adenomas with increased uptake as compared with the pituitary gland are considered positive. While in some cases this may lead to false positives if physiological pituitary uptake is higher than usual, a greater risk exists for false negatives when adenoma uptake is comparable to the applied limit. Additionally, in case of heterogeneous SSTR expression within an NFA, the low resolution can induce underestimation of the activity due to the partial volume effect (Borson-Chazot et al. 1997; Bai et al. 2013).

The two- to threefold higher spatial resolution of PET/CT in combination with more accurate attenuation correction makes it the preferred functional imaging modality for smaller lesions such as pituitary adenomas. Furthermore, uptake quantification with SUVs allows for a more objective evaluation. Of note, physiological uptake in the normal pituitary is also evident with ^68^Ga-DOTATATE PET, which hinders a straightforward assessment of the sellar region (Aalbersberg et al. 2019; Shastry et al. 2010). In the present study, we used co-registration with high-resolution MRI to optimise localisation of radiopharmaceutical uptake in the macroadenoma versus pituitary tissue. Still, maintaining a clear margin between the adenoma and pituitary is necessary to avoid activity spillover and overestimation of adenoma uptake. We therefore decided to place a circular ROI within the adenoma instead of manual delineation of the adenoma boundaries.

Partial volume effects play a role in the underestimation of radiotracer uptake in lesions smaller than two to three times the PET system’s spatial resolution (full width at half maximum) (Bettinardi et al. 2014). In our series, however, adenoma size was at least 12 mm, and consequently, the partial volume effect in our study is negligible. This may explain why we did not observe a significant correlation between adenoma size and SUV_mean_. It is therefore also unlikely that the PET-negative cases are false negatives due to the partial volume effect.

It is important to note that ^68^Ga-DOTATATE, as compared with ^111^In-DTPA-octreotide, has about 100 times higher affinity for SSTR2, decreased affinity for SSTR3 and similar affinity for SSTR5 (Reubi et al. 2000). In this light, the high positivity rate in our study was unexpected based on the notion that SSTR3 is the most abundantly expressed subtype in NFA (Even-Zohar and Greenman 2018; Colao et al. 2008). However, over the years, in vitro studies examining the SSTR expression in NFA specimens have produced conflicting results, pointing to either SSTR2 (Ramírez et al. 2012), SSTR3 (Gabalec et al. 2015) or SSTR5 (Fusco et al. 2012) as the dominant subtype. Possible explanations for these inconsistencies include differences in the method (detection of mRNA expression or protein, antibody specificity, membranous or cytoplasmic staining), patient selection (only gonadotropin-expressing or histopathologically diverse NFA) and the inherent heterogeneous distribution of various SSTR subtypes in NFA tissue samples. In the largest sample studied thus far, SSTR1–3 mRNA was expressed in 100% and SSTR5 mRNA in 60% of 198 specimens, using quantitative real-time RT-PCR (Gabalec et al. 2015). Nonetheless, mRNA levels do not always equal protein expression or the presence of functional receptors. This could explain the discrepancies between in vitro and in vivo results, as correctly translated, folded and transported SSTR proteins are required for ligand binding and in vivo detection.

The major advantage of ^68^Ga-DOTATATE PET is that it reliably visualises SSTR expression in vivo*.* Furthermore, assessment of ^68^Ga-DOTATATE uptake in NFMA may help to predict clinical response to SSTR2 preferential SSA (Colao et al. 2008).

## Conclusion

We present the first report of ^68^Ga-DOTATATE PET performed in a series of NFMA patients, demonstrating in vivo SSTR expression in the vast majority of cases. The high positivity rate suggests the presence of functional SSTR in more NFMA patients than previously reported. This opens novel perspectives for trials with somatostatin analogues, especially if a positive ^68^Ga-DOTATATE PET scan is used to select patients for treatment.

## Data Availability

The datasets generated and/or analysed during the current study are not publicly available as the study is embedded in an ongoing clinical trial but are available from the corresponding author on reasonable request.

## Abbreviations

^111^In-DTPA-octreotide: Indium-111-labelled diethylenetriaminepentaacetic acid-octreotide
^68^Ga-DOTATATE: Gallium-68-labelled dodecanetetraacetic acid-[Tyr^3^]-octreotate
AMC: Academic Medical Center (Amsterdam UMC, University of Amsterdam)
Amsterdam UMC: Amsterdam University Medical Centres
CT: Computer tomography
LUMC: Leiden University Medical Center
MRI: Magnetic resonance imaging
NFA: Non-functioning adenoma
NFMA: Non-functioning macroadenoma
PET: Positron emission tomography
ROI: Region of interest
SPECT: Single-photon emission computed tomography
SSA: Somatostatin analogue
SSTR: Somatostatin receptor
SUV: Standard uptake value
VUmc: VU University Medical Center (Amsterdam UMC, VU University)
